# Water-in-salt electrolytes made saltier by Gemini ionic liquids for highly efficient Li-ion batteries

**DOI:** 10.1038/s41598-023-29387-1

**Published:** 2023-02-07

**Authors:** Aleksandar Tot, Leiting Zhang, Erik J. Berg, Per H. Svensson, Lars Kloo

**Affiliations:** 1 Applied Physical Chemistry, Department of Chemistry, KTH Royal Institute of Technology, SE-10044 Stockholm, Sweden; 2 Department of Chemistry – Ångström Laboratory, Uppsala University, SE-751 21 Uppsala, Sweden; 3 Chemical and Pharmaceutical Development, RISE Research Institutes of Sweden, SE-151 36 Södertälje, Sweden

**Keywords:** Energy, Batteries

## Abstract

The water-in-salt electrolytes have promoted aqueous Li-ion batteries to become one of the most promising candidates to overcome safety concerns/issues of traditional Li-ion batteries. A simple increase of Li-salt concentration in electrolytes can successfully expand the electrochemical stability window of aqueous electrolytes beyond 2 V. However, necessary stability improvements require an increase in complexity of the ternary electrolytes. Here, we have explored the effects of novel, Gemini-type ionic liquids (GILs) as a co-solvent systems in aqueous Li[TFSI] mixtures and investigated the transport properties of the resulting electrolytes, as well as their electrochemical performance. The devices containing pyrrolidinium-based GILs show superior cycling stability and promising specific capacity in the cells based on the commonly used electrode materials LTO (Li_4_Ti_5_O_12_) and LMO (LiMn_2_O_4_).

## Introduction

The growing demand for rechargeable batteries with high performance based on sustainable materials combined with a high level of safety has directed research towards water-containing electrolytes^1–4^. Initially, aqueous electrolytes were avoided due to the low energy density, caused by the narrow electrochemical window of water itself^5^. The water-splitting issue was successfully overcome by the breakthrough discovery of Suo et al*.* where highly concentrated aqueous electrolytes were applied^6^. The salt concentrations are in fact so high, that they can be regarded as room-temperature hydrated molten salts. In such water-in-salt electrolytes (WISEs), most commonly based on lithium bis(trifluoromethanesulfonyl)imide (Li[TFSI]), the water activity decreases because of the strong interaction between water and lithium cations which is a consequence of reduced availability of “free” water^6–9^. In particular, the high concentration of salt directly dictates the thermodynamic state of water molecules, as well as of the salt anions, thus influencing their electrochemical behaviour. Recently, the importance of thermodynamic effects for the stability of WISEs have been more rigorously addressed. The strong coordination of water molecules to the lithium ions strengthens the intra-molecular O–H bond due to the absence of neighbouring water molecules, which as a consequence influences the electrochemical stability^10,11^. Moreover, there may also exist indirect kinetic effects relating to the formation of a solid-electrolyte interphase (SEI) at the anode^6,12^. In this context, the use of [TFSI]^–^ as anion, which strongly interacts with Li^+^ especially at such a high concentrations, was proven to favour the formation of an anion-derived SEI^13^. Synergies of these effects were claimed to result in an enhanced electrochemical stability window (ESW) of ≈2.5 V for WISEs in lithium-ion batteries (LIBs)^4,6,14^. However, a further increase of the ESW was observed to be limited by the solubility of lithium salts, which in turn also caused unwanted crystallization problems due to saturation problems^9,15^. As a potential solution, the introduction of multiple salts or co-solvents into the WISEs was successfully applied. This approach was driven by the idea to further reduce the water activity by forming a water-deficient solvation shell around the lithium cations and to promote the formation of an SEI. The extensive research in the field of the bi-salt approach and eutectic mixtures, introducing the combination of lithium salts with various perfluorinated anions, allowed solubilities up to 60 mol of salt per kg of water (60 m), and as a result, the ESW was boosted to > 3.5 V^4,16–18^. However, the viscosity of this type of fluid is quite high causing more sluggish mass transport, and thus reduced charge transport. Subsequently, different research groups have successfully expanded the cathodic voltage limit and increased the solubility of lithium salts by using co-solvents, such as dimethyl carbonate^19^, acetonitrile^20^, and tetraethylene glycol dimethyl ether^21^. Even though the resulting electrolytes are non-flammable, the manufacturing process still faces the problems of volatility and flammability of the co-solvents used, which constitute a major drawback for the implementation of this strategy in durable LIBs.

Recently, the advantage of the non-flammable and non-volatile nature of ionic liquids (ILs) was employed in the co-solvent strategy in WISEs. Chen et al*.* introduced the concept of lithium-ion salts in humid ionic liquids, where a successful expansion of the ESW was accomplished for two hydrophobic ILs (1-butyl-3-methylimidazolium bis(trifluoromethanesulfonyl)imide and 1-methyl-1-propylpyrrolidinium bis(trifluoromethanesulfonyl)imide)^11^. Furthermore, the use of the triethylmethylammonium [TFSI] facilitated a very high solubility of Li[TFSI] (63 m) and stable cycling in an operational LIB with suitable electrode materials^22^. In the work of Becker et al*.* (1-ethyl-3-methylimidazolium)[TFSI]/[TFO] ([TFO^-^] being the trifluorosulfonate anion) was introduced in Li[TFSI]- and Li[TFO]-based WISEs^23^. This strategy increased the solubility of the lithium-ion salts significantly, and at the same time allowed successful cycling of complete LIB cells with adapted electrode materials for over 500 charge/discharge cycles. The same research group also altered the activity of the water molecules in the electrolytes by introducing an additional co-solvent in the form of succinonitrile to form a quaternary system showing a high mobility of lithium ions and high stability during over 250 cycles^24^.

In the present work, the possibility to design ionic liquids to enhance specific physical and chemical properties has been further explored in order to more efficiently control the chemical activity of water. Instead of using commercially available ILs, we designed and synthesized completely new, so-called Gemini-type ionic liquids (GILs)^25^ and investigated them as the main components in a new generation of WISEs. This strategy was based on the use of two electrochemically stable, aliphatic cations as the cores for substitution, piperidinium, and pyrrolidinium, interlinked by polyethylene glycol (PEG) spacers. This class of GILs offers a combination of intrinsic high chemical stability, low viscosity and excellent solvent properties with respect to salts and water. The GILs involving ether-containing spacers display structural similarity to PEG-based compounds, which have shown superior electrochemical and cycling stability^26,27^. Moreover, the potential use of ILs in the WISEs is hampered by high viscosity of the resulting mixtures, which may be counter-acted by the synthetic strategy of using ether-containing alkyl chains. The choice of [TFSI]^–^ as anion in the GILs is based on the serendipitous decompositional formation of favorable SEIs and the tendency to exhibit water-blocking behavior at the electrode/electrolyte interface by the anion^23^. Numerous studies have demonstrated these benefits, and [TFSI]^–^ is considered to be the ideal candidate as anion in WISEs^5,6,23,28^. Moreover, [TFSI]^–^ and the employed cations can be systematized as structure-breaking (chaotropic) ions, which will play a significant role in modifying the chemical activity of water influencing the electrochemical stability of the electrolytes^13,29^. The chaotropic properties of the GILs can be further tuned by side-chain substitution. In spite of lower ionic mobility, the GILs offer significantly wide ESWs and high electrochemical stability of the resulting LIBs. Using the GILs, we have been able to demonstrate devices with highly competitive energy capacity and superior stability without an increase of the Li-salt concentration; an otherwise typical approach used in this type of WISEs. Another advantage of these systems is the possibility to assemble cells in ambient air, which could significantly reduce the costs of a manufacturing process. Moreover, our systems demonstrate compatibility and excellent charge/discharge cycling performance together with commercial LTO (Li_4_Ti_5_O_12_) and LMO (LiMn_2_O_4_) electrode materials, without the need for any additional protective coatings.

## Results and discussion

**Figure 1:**
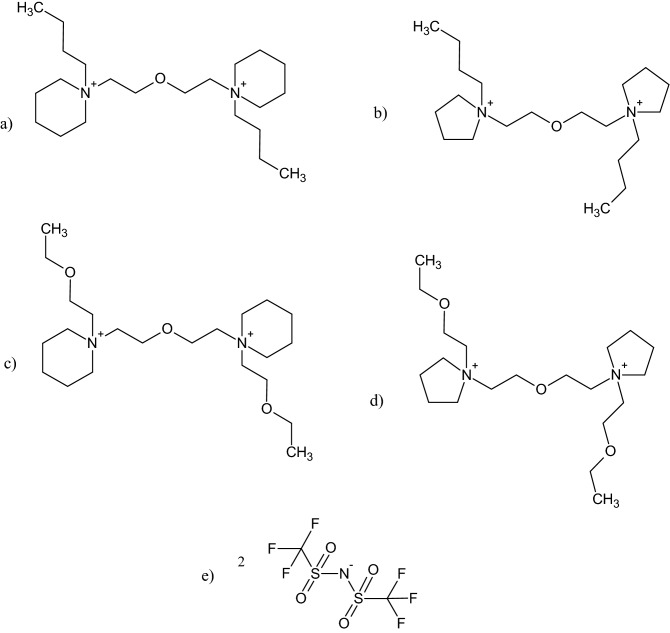
The ionic components of the GILs studied in this work: (**a**) 1,1–2-oxabutane-bis-1-butylpiperidinium, (C_4_Pip-C_2_OC_2_-C_4_Pip)^2+^; (**b**) 1,1–2-oxabutane-bis-1-butylpyrrolidinium, (C_4_Pyr-C_2_OC_2_-C_4_Pyr)^2+^; (**c**) 1,1–2-oxabutane-bis-1-(2-oxabutyl)piperidinium, (C_2_OC_2_Pip-C_2_OC_2_- C_2_OC_2_Pip)^2+^; (**d**) 1,1–2-oxabutane-bis-1-(2-oxabutyl)pyrrolidinium, (C_2_OC_2_Pyr-C_2_OC_2_- C_2_OC_2_Pyr)^2+^; (**e**) bis(trifluoromethylsulfonyl) imide, [TFSI]^–^.

In the present study, the typical WISE is based on a novel, dicationic pyrrolidinium and piperidinum IL (Fig. 1, Table 1). The high electrochemical stability of the pyrrolidinium and piperidinium ILs was a crucial factor for selecting these cations instead of the more common aromatic ones, such as the imidazolium cation. This particularly refers to the reductive stability, where imidazolium-based ILs, such as 1-butyl-3-methylimidazolium bis(trifluoromethanesulfonyl)imide exhibits a reductive potential of about – 2.6 V vs. Li/Li^+^^30,31^, whereas pyrrolidinium and piperidinium analogues display reductive potentials of – 2.92 V and – 3.55 V vs. Li/Li^+^, respectively^31^.

**Table 1:** Water content and yield of synthesized ionic liquids.

Ionic liquid	Yield (%)	Water content (ppm)
(C_4_Pip-C_2_OC_2_-C_4_Pip)[TFSI]_2_	85	6.4
(C_4_Pyr-C_2_OC_2_-C_4_Pyr)[TFSI]_2_	93	10.2
(C_2_OC_2_Pip-C_2_OC_2_- C_2_OC_2_Pip)[TFSI]_2_	90	7.8
(C_2_OC_2_Pyr-C_2_OC_2_- C_2_OC_2_Pyr)[TFSI]_2_	79	12.9

In this work, all ternary electrolyte systems were prepared using the same molar ratio of Li[TFSI], corresponding GIL, and water (1:1:3), and all obtained results are compared to the non-IL-containing WISE in the ratio of 1:3 (Li[TFSI]: water). This ratio between lithium salt and water was used to avoid possible oversaturation considering that the preparation process of the electrolyte mixtures involved no initial heating^9^.

### Viscosity and conductivity

**Figure 2:**
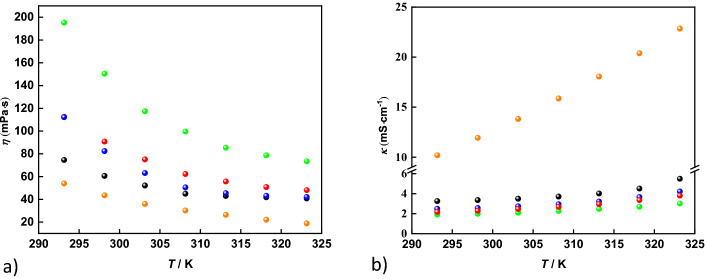
The temperature-dependent experimental viscosities (**a**) and electrical conductivities (**b**) for the investigated systems. Black – Li[TFSI]_(1)_:(C_2_OC_2_Pyr-C_2_OC_2_-C_2_OC_2_Pyr)[TFSI]_2(1)_:H_2_O_(3)_; red—Li[TFSI]_(1)_:(C_4_Pyr-C_2_OC_2_-C_4_Pyr)[TFSI]_2(1)_:H_2_O_(3)_, blue – Li[TFSI]_(1)_:(C_2_OC_2_Pip-C_2_OC_2_-C_2_OC_2_Pip)[TFSI]_2(1)_:H_2_O_(3)_; green—Li[TFSI]_(1)_:(C_4_Pip-C_2_OC_2_-C_4_Pip)[TFSI]_2(1)_:H_2_O_(3)_; orange—Li[TFSI]_(1)_:H_2_O_(3)_.

The presence of GILs in the aqueous Li[TFSI] mixture increases the viscosity of the resulting electrolytes (Fig. 2a). The addition of GILs to an Li[TFSI]_(1)_:H_2_O_(3)_ electrolyte raises the viscosity at 298 K from 43 mPa·s up to 150 mPa·s for the system Li[TFSI]_(1)_:(C_4_Pip-C_2_OC_2_-C_4_Pip)[TFSI]_2(1)_:H_2_O_(3)_. However, by comparing the results of all investigated electrolytes, it can be concluded that the addition of the pyrrolidinium cation with the ether-containing side-chain and spacer ((C_2_OC_2_Pyr-C_2_OC_2_-C_2_OC_2_Pyr)[TFSI]_2_) only increases the viscosity to 61 mPa·s at 298 K. Overall, the electrolytes containing the pyrrolidinium GILs show lower viscosities as compared to the piperidinium analogues. Also, the presence of an oxabutyl group as side-chain has a significant impact on the resulting viscosity, to a larger extent than changing the cation core, since the piperidinium GILs with ether-containing side-chains show lower viscosities as compared to the pyrrolidinium GILs with butyl side-chains. Comparing these viscosities with those reported in the work of Becker et al*.* (Table 2)^23^, our systems display slightly higher viscosities than a mixture of Li[TFSI]/(EMIm)[TFSI]/H_2_O (44 mPa·s at 298 K) in a corresponding molar ratio.

**Table 2:** The properties and electrochemical performance summary of systems investigated in this work and in the work by Becker et al.^23^.

System	Viscosity (mPa·s) at 298 K	Conductivity (mS·cm^−1^) at 298 K	Discharge specific capacity (mA·h·g^-1^)
Li[TFSI]_(1)_:(C_2_OC_2_Pyr-C_2_OC_2_-C_2_OC_2_Pyr)[TFSI]_2(1)_:H_2_O_(3)_	60.6	3.4	90
Li[TFSI]_(1)_:(C_4_Pyr-C_2_OC_2_-C_4_Pyr)[TFSI]_2(1)_:H_2_O_(3)_	90.8	2.6	89
Li[TFSI]_(1)_:(C_2_OC_2_Pip-C_2_OC_2_-C_2_OC_2_Pip)[TFSI]_2(1)_:H_2_O_(3)_	82.4	2.3	69
Li[TFSI]_(1)_:(C_4_Pip-C_2_OC_2_-C_4_Pip)[TFSI]_2(1)_:H_2_O_(3)_	150.5	2.0	67
Li[TFSI]_(1)_:(C_2_Im) [TFSI]_(1)_:H_2_O_(3)_^23^	44.2	6.7	60

The electrical conductivities of the electrolytes after the addition of the GILs are significantly lower as compared to aqueous Li[TFSI] (11.9 mS·cm^−1^ at 298 K), which can be observed in Fig. 2b. The least conductive system includes (C_4_Pip-C_2_OC_2_-C_4_Pip)[TFSI] with an electrical conductivity close to 2 mS·cm^–1^ at 298 K. As expected, the conductivities are strongly correlated to the corresponding viscosities with the best performance shown by the system containing the (C_2_OC_2_Pyr-C_2_OC_2_-C_2_OC_2_Pyr)[TFSI]_2_ GIL with 3.4 mS·cm^–1^ at 298 K. Comparing the results of the systems containing different GILs, it can be concluded that the electrolytes containing pyrrolidinium-based GILs are more conductive and that the introduction of an ethyl group in the side-chain increases the conductivity by ≈30% as compared to the analogues with a butyl side-chain. The most promising electrolyte still only shows half the conductivity compared to the best ones in Becker's work,^23^ 6.7 mS·cm^–1^ at 298 K for the electrolyte containing (EMIm)[TFSI]. However, when evaluating the viscosity and conductivity of the systems studied in our work, it is important to emphasize that although the overall Li-salt concentration in our systems can be formulated as about 19 mol/kg, the total salt concentration is about 40 mol/kg using the conventional annotation with respect to water. It is also important to mention that 1 mol of cation contributes with 2 mol of [TFSI]^–^, leading to a higher molar concentration of anions in our systems in comparison to conventional monocationic salts or ILs. Therefore, the obtained transport results seem very reasonable.

### Electrochemical performance

**Figure 3:**
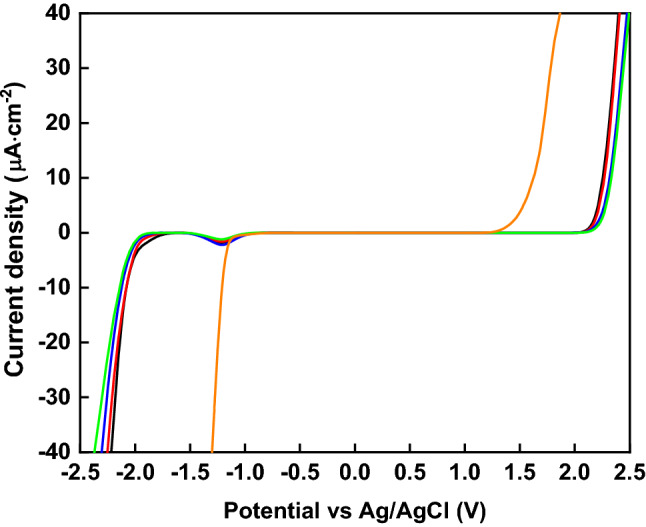
The ESWs of the investigated electrolytes using stainless steel current collectors. Black—Li[TFSI]_(1)_:(C_2_OC_2_Pyr-C_2_OC_2_-C_2_OC_2_Pyr)[TFSI]_2(1)_:H_2_O_(3)_; red—Li[TFSI]_(1)_:(C_4_Pyr-C_2_OC_2_-C_4_Pyr)[TFSI]_2(1)_:H_2_O_(3)_, blue—Li[TFSI]_(1)_:(C_2_OC_2_Pip-C_2_OC_2_-C_2_OC_2_Pip)[TFSI]_2(1)_:H_2_O_(3)_; green—Li[TFSI]_(1)_:(C_4_Pip-C_2_OC_2_-C_4_Pip)[TFSI]_2(1)_:H_2_O_(3)_; orange—Li[TFSI]_(1)_:H_2_O_(3)_.

Despite the transport properties described above, this type of highly concentrated electrolyte benefits the most from better electrochemical stability upon the addition of the GILs. The electrochemical stability of the novel electrolytes was investigated by linear-sweep voltammetry (Fig. 3). The experiments were performed using stainless steel current collectors. A low current–density cut-off (40 μA·cm^−2^) was used in all measurements to offer a reliable ESW, as has been proposed by Kühnel et al*.*^32^ The presence of GILs induces a wide ESW of 3.7 V or higher, which represents a significant improvement of ≈1.5 V as compared to Li[TFSI]_(1)_:H_2_O_(3)_ (ESW≈2.35 V). The reductive stabilities are excellent for the electrolytes containing the piperidinium GILs (− 1.88 V and − 1.96 V vs. Ag/AgCl for (C_2_OC_2_Pip-C_2_OC_2_-C_2_OC_2_Pip)[TFSI]_2_ and (C_4_Pip-C_2_OC_2_-C_4_Pip)[TFSI]_2_, respectively) in comparison with the pyrrolidinium-based GILs (− 1.75 V and − 1.85 V vs. Ag/AgCl for (C_2_OC_2_Pyr-C_2_OC_2_-C_2_OC_2_Pyr)[TFSI]_2_ and (C_4_Pyr-C_2_OC_2_-C_4_Pyr)[TFSI]_2_). The oxidative stability limits were found to be similar for all electrolytes with the same type of IL cation core, irrespective of the different alkyl substituents introduced. Again, piperidinium-containing electrolytes show better oxidative stability as compared to electrolytes based on pyrrolidinium. The origin of the small peak that is visible in the voltammograms at ≈− 1.0 V vs. Ag/AgCl in all the electrolyte systems is unclear and debated in the literature. Two possibilities have been proposed: (1) the reduction of [TFSI]^–^, which represents a process that is essential to the formation of an anion-induced SEI^33^, (2) the reduction of water, leading to the formation of hydroxyl groups that are responsible for nucleophile attack on [TFSI]^–^ and a resulting formation of LiF^34^. The high concentrations of anions in our systems, due to the presence of the dications in the GILs, have a dual effect: (1) to form a more stable, compact SEI layer due to the decomposition of the [TFSI]^–^ anion; (2) to interact more effectively with the Li-ions. Therefore, the high concentrations of [TFSI]^–^ boost the electrochemical stability, which is further improved by the presence of the chaotropic GIL cations that improve the water stability by disrupting the intrinsic water structure. The systematic correlation between battery performance and structure-breaking/making cations as electrolytes in WISE has not previously been performed, offering only information about the influence of a structure-breaking tendency of anions on the electrochemical stability of WISE^13^. Therefore, the variation of the cation structure-breaking properties performed in this work provide additional insights regarding the correlation between electrochemical stability and the reduction in chemical activity of water. The wide ESWs in the investigated electrolytes should be compatible with a Li_4_Ti_5_O_12_ (LTO) anode (≈ − 1.5 V vs. Ag/AgCl in concentrated electrolytes) and LiMn_2_O_4_ (LMO) cathode (≈1.2 V vs. Ag/AgCl in concentrated electrolytes)^35^. Therefore, these electrode materials were used also to investigate the charge/discharge cycling performance.

**Figure 4:**
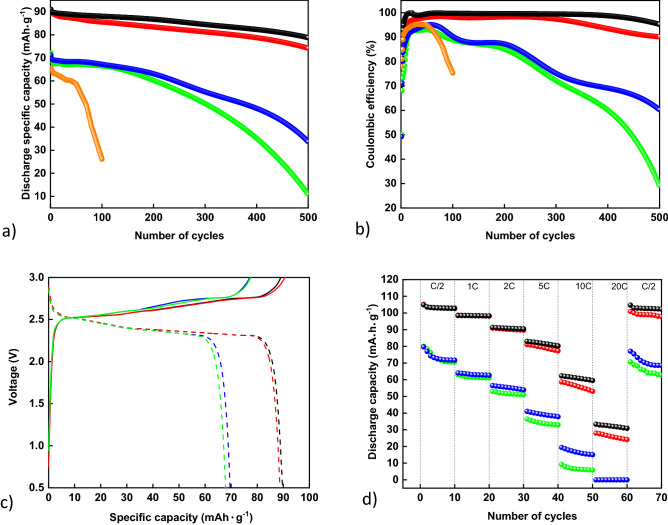
Electrochemical performance of LTO/LMO-based full battery cells. (**a**) Cycling stability during discharging, (**b**) Coulombic efficiency, (**c**) Voltage profiles for the 10th cycle, (**d**) Rate performance. Black—Li[TFSI]_(1)_:(C_2_OC_2_Pyr-C_2_OC_2_-C_2_OC_2_Pyr)[TFSI]_2(1)_:H_2_O_(3)_; red—Li[TFSI]_(1)_:(C_4_Pyr-C_2_OC_2_-C_4_Pyr)[TFSI]_2(1)_:H_2_O_(3)_, blue—Li[TFSI]_(1)_:(C_2_OC_2_Pip-C_2_OC_2_-C_2_OC_2_Pip)[TFSI]_2(1)_:H_2_O_(3)_; green—Li[TFSI]_(1)_:(C_4_Pip-C_2_OC_2_-C_4_Pip)[TFSI]_2(1)_:H_2_O_(3)_; orange—Li[TFSI]_(1)_:H_2_O_(3)_.

The charge/discharge cycling performance of full cells based on the electrode materials LTO/LMO together with the novel electrolytes are shown in Fig. 4, including a comparison to an aqueous Li[TFSI] electrolyte. The cells were cycled between 0.5 V and 3.0 V at a rate that allows discharging of the battery in one hour (1C). The voltage profiles of full cells for selected cycles are presented in Figs. S5–S8 in the Supporting information. The specific capacity was calculated in respect to the active mass of LTO. The high electrochemical stability noted for electrolytes containing the new GILs translates to better cycling stability as compared to aqueous Li[TFSI] electrolytes (Fig. 4a,b). The main reason can be linked to the enhanced reductive stability provided by the addition of the GILs. The literature suggests that the LTO electrode material is slightly outside the ESW in traditional WISEs, leading to fast decline in Coulombic efficiency (CE) caused by the amount of “free water” available in the electrolyte^23,24^. The maximum CE for the electrolyte Li[TFSI]_(1)_:H_2_O_(3)_ obtained in this work is close to 95%, which is reached after 10 cycles. However, both charge- and discharge-specific capacities fade rapidly upon cycling, with a significantly faster decay in the charging capacity. After 50 cycles, both charging and discharging capacities for LIBs based on the Li[TFSI]_(1)_:H_2_O_(3)_ electrolytes are below 60 mA·h·g^–1^, while after 100 cycles the charging capacity falls below 35 mAh·g^–1^ and the discharging capacity below 25 mA·h·g^–1^. A similar trend was reported by Becker et al.^23^, with slightly longer battery lifetime for the GIL-free systems investigated in our work. The enrichment of aqueous Li[TFSI] with GILs increases the charge/discharge cycling lifetime of LTO/LMO-based battery cells, characterized by a fast decline in the charging capacity during initial cycling, while the discharging capacity is more stable during the initial cycles. The initial capacity decay in WISE-based LTO/LMO cells is commonly attributed to the consumption of lithium from a slightly oversized LMO electrode during the formation of the SEI at a high overpotential^22–24,35^.

A significant difference can be observed in the systems containing the pyrrolidinium-based GILs when compared to the piperidinium-based ones. LIBs based on both pyrrolidinium-based GILs show almost 30% higher charging and discharging capacity during cycling as compared to the corresponding piperidinium-containing ones. From the perspective of CE, the best-performing coin cells contain the Li[TFSI]_(1)_:(C_2_OC_2_Pyr-C_2_OC_2_-C_2_OC_2_Pyr)[TFSI]_2(1)_: H_2_O_(3)_ electrolyte, which retains at least 99.5% of the CE for minimum 354 cycles, with an average CE of 99.6% from cycle 6 to cycle 300. After 500 cycles, this battery system retained a CE of 95.2% and 86.5% of its initial discharge capacity. LIBs based on the electrolyte with the aliphatic side-chain attached to the pyrrolidinium backbone showed a similar performance, albeit not as good displaying an average CE of 98.0% in the first 300 cycles, a final CE of 90.2%, retaining 82.3% of the initial discharging capacity. On the other hand, the electrolytes enriched with the corresponding piperidinium-based GIL analogues display a faster decline in cycling performance, with a maximum CE close to 95% followed by a fast decrease after 100 cycles. The piperidinium-based systems retain only 47.4% (Li[TFSI]_(1)_:(C_2_OC_2_Pip-C_2_OC_2_-C_2_OC_2_Pip)[TFSI]_2(1)_:H_2_O_(3)_) and 15.1% (Li[TFSI]_(1)_:(C_4_Pip-C_2_OC_2_-C_4_Pip)[TFSI]_2(1)_:H_2_O_(3)_) of the initial discharging capacity after 500 cycles. Battery systems based on (C_4_Pip-C_2_OC_2_-C_4_Pip)[TFSI] exhibited the worst performance among the novel GILs used in this work, with an accelerated decrease of CE after 200 cycles. The difference in cycling performance between pyrrolidinium and piperidinum-containing electrolytes could be observed from a comparison of the voltage profiles for the 10th cycle (Fig. 4c). The curves show a characteristic discharge plateau at ≈2.50 V to ≈2.30 V and a charge voltage plateau from ≈2.50 V to ≈2.70 V, corresponding to the deintercalation/intercalation of Li^+^ from/into LMO/LTO^22–24,35^. The electrolytes with pyrrolidinium GILs exhibited larger charging/discharging overpotentials. Besides lower overpotentials of piperidinium-based electrolytes, a significant difference could be observed in the charging plateaus. The appearance of two different slopes in the case of the piperidinium electrolytes suggest that a potential side reaction could be undergoing, resulting in a lowering of the specific energy capacity and consequently the CE.

For the rate performance of GIL-containing electrolyte systems (Fig. 4d), 10 cycles at different discharge rates were performed. The experiments started with the slowest discharge rate, which allows 2 h (C/2) until full discharge of the battery. After 10 cycles at the stated rate, the current density was increased to allow discharge in one hour (1C), and this procedure was analogously expanded to 2C, 5C, 10C, and 20C, and was at the end of the study again returned to C/2 to check the system resilience (Fig. 4d). Again, electrolytes based on the pyrrolidinium GILs proved to be superior. The electrolytes based on the pipperidium-type GILs show a more significant decrease in the discharge-specific capacity using the higher current density (higher rates) until they stop working at 20C. Regarding pyrrolidinium-based electrolytes, the batteries based on Li[TFSI]_(1)_:(C_2_OC_2_Pyr-C_2_OC_2_-C_2_OC_2_Pyr)[TFSI]_2(1)_:H_2_O_(3)_ proved to be the most robust with respect to a change of the rate, being superior to systems based on the electrolyte Li[TFSI]_(1)_:(C_4_Pyr-C_2_OC_2_-C_4_Pyr)[TFSI]_2(1)_:H_2_O_(3)_ at higher rates. The best rate performance of the electrolyte Li[TFSI]_(1)_:(C_2_OC_2_Pyr-C_2_OC_2_-C_2_OC_2_Pyr)[TFSI]_2(1)_:H_2_O_(3)_ could be ascribed to its viscosity and conductivity, being the lowest and higest respectively, when compared to the other systems investigated. The batteries containing the less viscous systems show better resilience. Batteries based on Li[TFSI]_(1)_:(C_2_OC_2_Pyr-C_2_OC_2_-C_2_OC_2_Pyr)[TFSI]_2(1)_:H_2_O_(3)_ retrieve 99.9% of the initial discharge capacity at C/2, while the worst performing batteries consist of Li[TFSI]_(1)_:(C_4_Pip-C_2_OC_2_-C_4_Pip)[TFSI]_2(1)_:H_2_O_(3)_ showing only 84.3% of their initial discharge capacity.

Even though piperidinium-containing electrolytes show better electrochemical stability, their performance in operational batteries is worse. The larger ESW cannot compensate for the high viscosities and low ion mobility subsequently influencing the ultimate battery performance. However, the tuning of cation chaotropicity with the inclusion of ether-based side-chain results in superior device performance as compared to the alkyl-chain analogues. Indeed, ether-based side-chains decrease the viscosity of the systems. However, the influence of the more structure-affecting group on the water structure and resulting changes in the Li-ion-solvation cannot be neglected. Initial investigations the coordination of [TFSI]^–^ to Li^+^ exploiting the Raman band of the anion in the range 740–760 cm^-1^, show a shift to lower wavenumbers after addition of the GIL (Fig. S9). This band represents the breathing mode of vibration of [TFSI]^–^, typically found at 750 cm^–1^ when the anion is fully coordinated to a metal ion and at 740 cm^–1^ when [TFSI]^–^ is non-coordinated^6^. Therefore, a decrease of the Raman wavenumber after the addition of GILs indicates a change in Li-ion solvation structure as compared to the Li-salt-water mixture. Molecular dynamic simulations show the formation of nano-size domains in the systems containing GILs, in contrast to the non-GIL systems, most likely formed bacuse of expulsion of the water molecules by the hydrophobic parts of the ionic liquids; like a nono-size phase separation. A more comprehensive investigation of the Li-ion solvation environment will be a focus of future work. Moreover, the pyrrolidinium GILs presented in this work demonstrate a promising alternative in designing highly stable aqueous lithium-ion batteries, avoiding the typical approach of increasing the Li-salt concentrations (> 21 mol/kg). Compared to the LTO/LMO full cells with IL-containing WISEs (Table 2)^23^, our systems show higher discharge specific energy capacity by almost 10 mAh·g^–1^. Further improvement of GIL-type ternary electrolytes and subsequently device performance should be expected due to the unlimited design potential of novel ionic liquids. However, since the design and compositional space of electrolytes are immense, we also have ongoing work utilizing robotized combinatorial screening to accelerate the discovery of efficient, safe and sustainable water-based electrolytes. Finally, the task-specific nature of GILs can present a promising and efficient way to distinguish thermodynamic and kinetic effects responsible for the enlargement of ESWs in WISE-type battery cells.

## Methods

### Synthesis of ionic liquids

The syntheses were performed in three steps similar for all ionic liquids (Scheme S1). In the first phase, 0.1 mol of pyrrolidine (Sigma Aldrich, CAS number: 123-45-1, purity: 99%) or piperidine (Sigma Aldrich, CAS number: 110-89-4, purity: 99%) was dissolved in acetone, and mixed with an equimolar (0.1 mol) amount of 2-chloroethyl ethyl ether (Sigma Aldrich, CAS number: 628-34-2, purity: 99%) or 1-chlorobutane (Sigma Aldrich, CAS number:109-69-3, purity: 99%) and solid potassium carbonate (0.2 mol). The mixture was stirred for 48 h at 50 °C under nitrogen. The unreacted material was subsequently removed along with the solvent, and the product obtained was dried for 24 h under vacuum. 2 molar equivalents of the obtained N-alkylpyrrolidine or N-alkylpiperidine were mixed with 1 molar equivalent of bis(2-chloroethyl) ether (Sigma Aldrich, CAS number: 111–44-4, purity: 99%). Isopropanol was used as solvent and the mixture was stirred at 80 °C under reflux for 72 h. After removal of the solvent, the product was purified five times by recrystallization in acetone. The obtained halide GILs were dried under vacuum over phosphorous pentoxide for 2 days. The purity was determined by argentometric potentiometric titration, and the amount of water was determined by Karl-Fisher titration.

In the last step, the chloride anion was exchanged for [TFSI]^–^. One molar equivalent of ionic liquid was dissolved in water and a 2 molar equivalent of Li[TFSI] was added. The solution was vigorously stirred for 24 h at room temperature and resulted in the separation of two phases. The aqueous phase, containing LiCl(aq), was removed, and the resulting ionic liquids were further washed with dichloromethane. After removing dichloromethane, and drying, the water content, as well as the amount of chloride, was determined as described above. The yield and water content in all ionic liquids were determined and are presented in Table 1. The structure was confirmed by NMR and FT-IR spectroscopy (Figs. S1–S4). Electrospray ionization mass spectra were acquired with a Finnigan LCQ ion trap mass spectrometer (Finnigan, San Jose, CA). Sample solutions in 50:50 methanol/water were directly infused into the mass spectrometer with a continuous flow of 5 μL/min using a syringe pump. The instrument was set in positive mode, and the LCQ ion source was operated at 5 kV. Nitrogen was used as nebulizing gas, and helium was used for damping and as collision gas.

### Electrolyte preparation

Lithium bis(trifluoromethanesulfonyl)imide, Li[TFSI], was purchased from Sigma Aldrich (CAS number: 90076-65-6, initial purity of 99.99%). Before the preparation of the electrolytes, Li[TFSI] was dried and stored under vacuum, with subsequent analysis of the water content by Karl-Fisher titration. The high-purity water (Millipore Milli-Q) was degassed and purged with argon before use.

### Viscosity and conductivity investigations

The viscosities were determined using a Brookfield CAP 2000 + viscometer. A Mettler-Toledo Seven Excellence™ conductometer S700 with platinum electrodes was used for the electrical conductivity experiments. The analyses were performed in a Pyrex-type of cell with an external water flow to retain a constant temperature using a Lauda thermostat. The conductivity cell constant was pre-determined using a 0.01 M aqueous KCl standard solution and validated at all temperatures investigated.

### Electrode preparation

Active materials, lithium titanate (LTO) and lithium manganate (LMO) were purchased from Sigma Aldrich, as well as the binder polyvinylidene fluoride (PVDF). The conductive carbon material SuperC65 was bought from MSE Suppliers. For the preparation of the electrode slurry, the active material, conductive carbon, and the binder were mixed in a mass ratio of 8:1:1. PVDF was pre-dissolved in N-methyl pyrrolidone to form a 5% solution. The viscous slurry was coated onto the stainless-steel current collector and dried for 6 h at 80 °C in air, and subsequently for 6 h at 80 °C under vacuum. The electrodes were punched in a diameter of 13 mm. The loading mass of LTO was 2.4 mg, while the corresponding mass of LMO was 3.1 mg.

### Battery assembly

The stability window of the electrolytes was determined in a Swagelok T-cell assembly, using stainless steel as working and counter electrodes, and a miniature Ag/AgCl electrode (eDAQ) was used as a reference electrode. Two-electrode full battery cells were assembled using CR2032 stainless-steel coin cells with Whatman GF/D glass microfiber separators (d = 15 mm). The electrolyte volume used was 55 μL. All cells were assembled in an ambient atmosphere and allowed to equilibrate for 24 h before characterization.

### Electrochemical characterization

Linear sweep voltammetry was performed in the Swagelok T-cells using a scan rate of 0.1 mV/s and an AutolabPG12 potentiostat. The galvanostatic cycling was performed at a Neware battery station using coin-type cells. The voltage cut-off was set to 0.5 and 3.0 V. The current density was adjusted according to the mass of the LTO electrode. The electrochemical characterization was performed without control of the temperature. All the presented data are the averages of 8 coin cells.

## Supplementary Information

Supplementary Information.

## Data Availability

Most data supporting the findings in this study are available from the manuscript and its Supplementary Information. Raw data sets can be obtained from the corresponding or first author on request.
