# Supplementary material for: Water-in-salt electrolytes made saltier by Gemini ionic liquids for highly efficient Li-ion batteries
Source: Sci Rep. 2023 Feb 7;13:2154. doi: 10.1038/s41598-023-29387-1 (PMC9905052; doi:10.1038/s41598-023-29387-1)
Supplement: Supplementary file 1 — Supplementary Information. [file 41598_2023_29387_MOESM1_ESM.docx]

**Water-in-salt electrolytes made saltier by Gemini ionic liquids for highly efficient Li-ion batteries**

Aleksandar Tot^a^, Leiting Zhang^b^, Erik J. Berg^b^, Per H. Svensson^c^, Lars Kloo^a^

*^a^Applied Physical Chemistry, Department of Chemistry, KTH Royal Institute of Technology, Stockholm, SE-10044, Sweden*

*^b^Department of Chemistry – Ångström Laboratory, Uppsala University, Uppsala, SE-751 21, Sweden*

*^c^**Chemical and Pharmaceutical Development,* *RISE Research Institutes of Sweden, Södertälje, SE-151 36, Sweden*

-Electronic Supplementary Information-

a)

b)

**Scheme S1.** Synthesis of piperidinium (a) and pyrrolidinium (b) ionic liquids.

R=(–C_4_H_9_) or (–C_2_H_4_–O–C_2_H_5_)


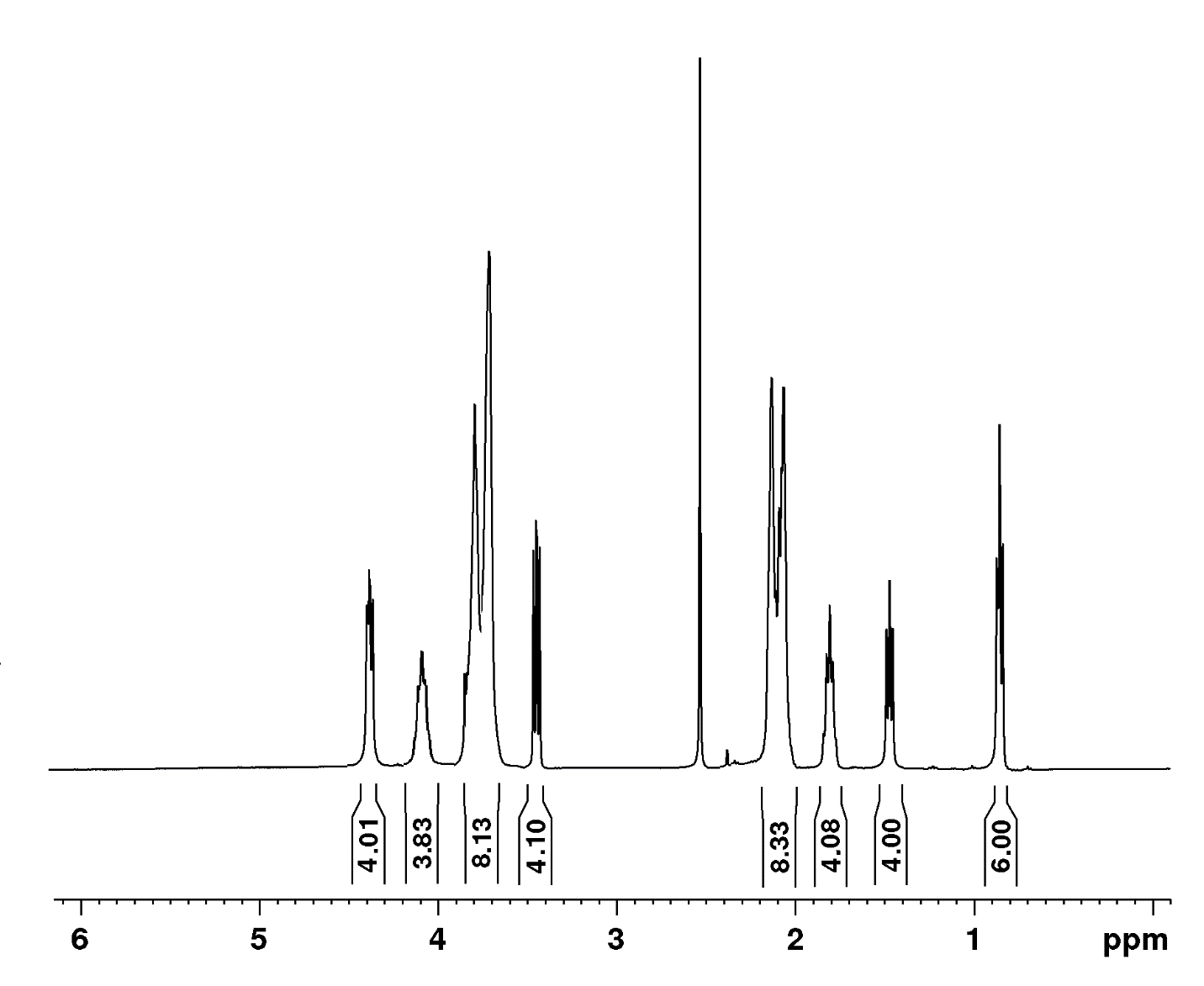


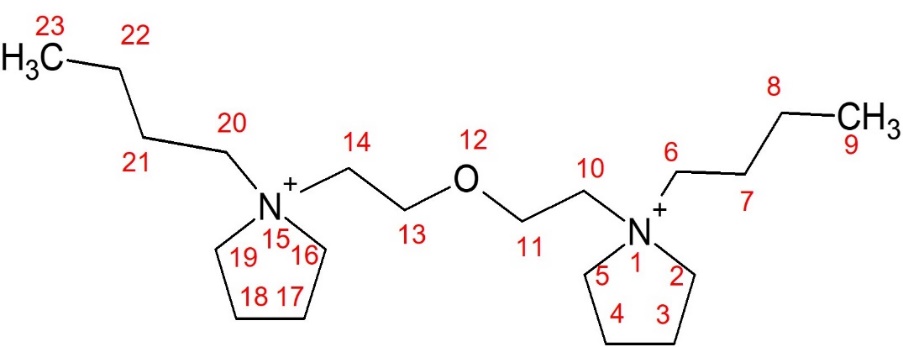


**Figure S1a.** ^1^H NMR spectra of (C_4_Pyr-C_2_OC_2_-C_4_Pyr)[TFSI]_2_

^1^H (d_6_-DMSO): 0.87 (6H, C9 and C23), 1.45 (4H, C8 and C22), 1.83 (4H, C7 and C20), 2.01-2.15 (8H, C3, C4, C17, C18),3.48 (4H, C6, C20), 3.68-3.88 (8H, C2, C5, C16, and C19), 4.01 (4H, C10 and C14), 4.28 (4H, C11 and C13)


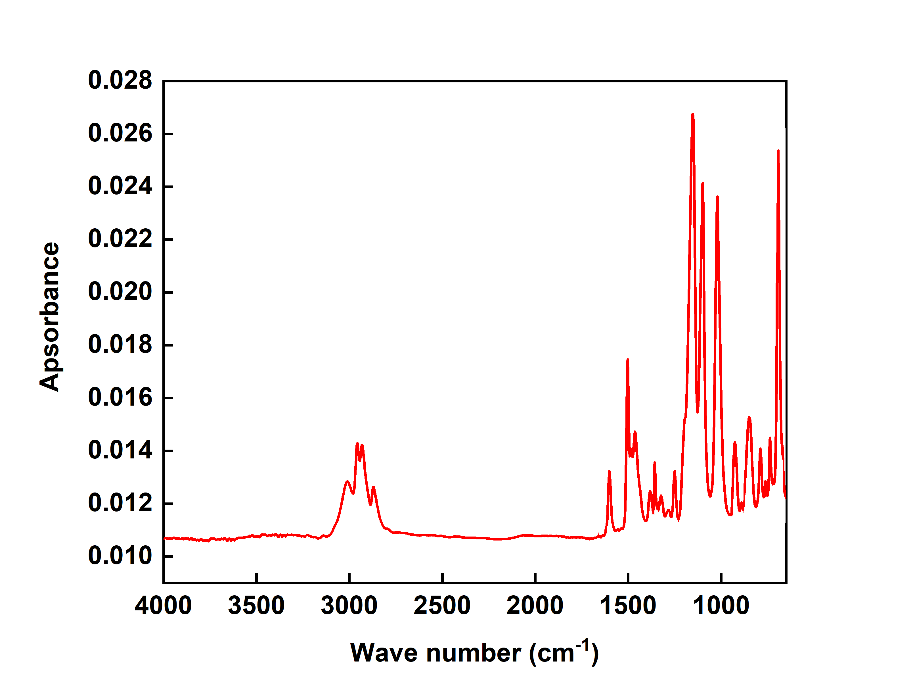


**Figure S1b.** FT-IR spectrum of (C_4_Pyr-C_2_OC_2_-C_4_Pyr)[TFSI]_2_

IR assignment: 3002 cm^-1^ C-H stretch mode in CH_3_; 2943, 2921 and 2855 cm^-^1 C-H stretch modes in CH_2_ of pyrrolidinium ring; 1596 cm^-1^ N-C bend mode in pyrrolidinium; 1465 cm^-1^ CH_3_ scissor mode (connected with the N-atom); 1450 cm^-1^ asymmetric deformation mode of the butyl chain; 1200 cm^-1^ symmetric stretch mode of C-O-C; 1174 cm^-1^ symmetric stretch mode in SO_2_; 1134 cm^-1^ asymmetric strech mode of C-O-C; 923 cm^-1^ pyrrolidinium ring bend mode; 791 cm^-1^ stretch mode of S-N-S; 765 cm^-1^ stretch mode of C-S; 754 cm^-1^ stretch mode in CF_3_

**Figure S1c.** Mass spectrum of (C_4_Pyr-C_2_OC_2_-C_4_Pyr)[TFSI]_2_


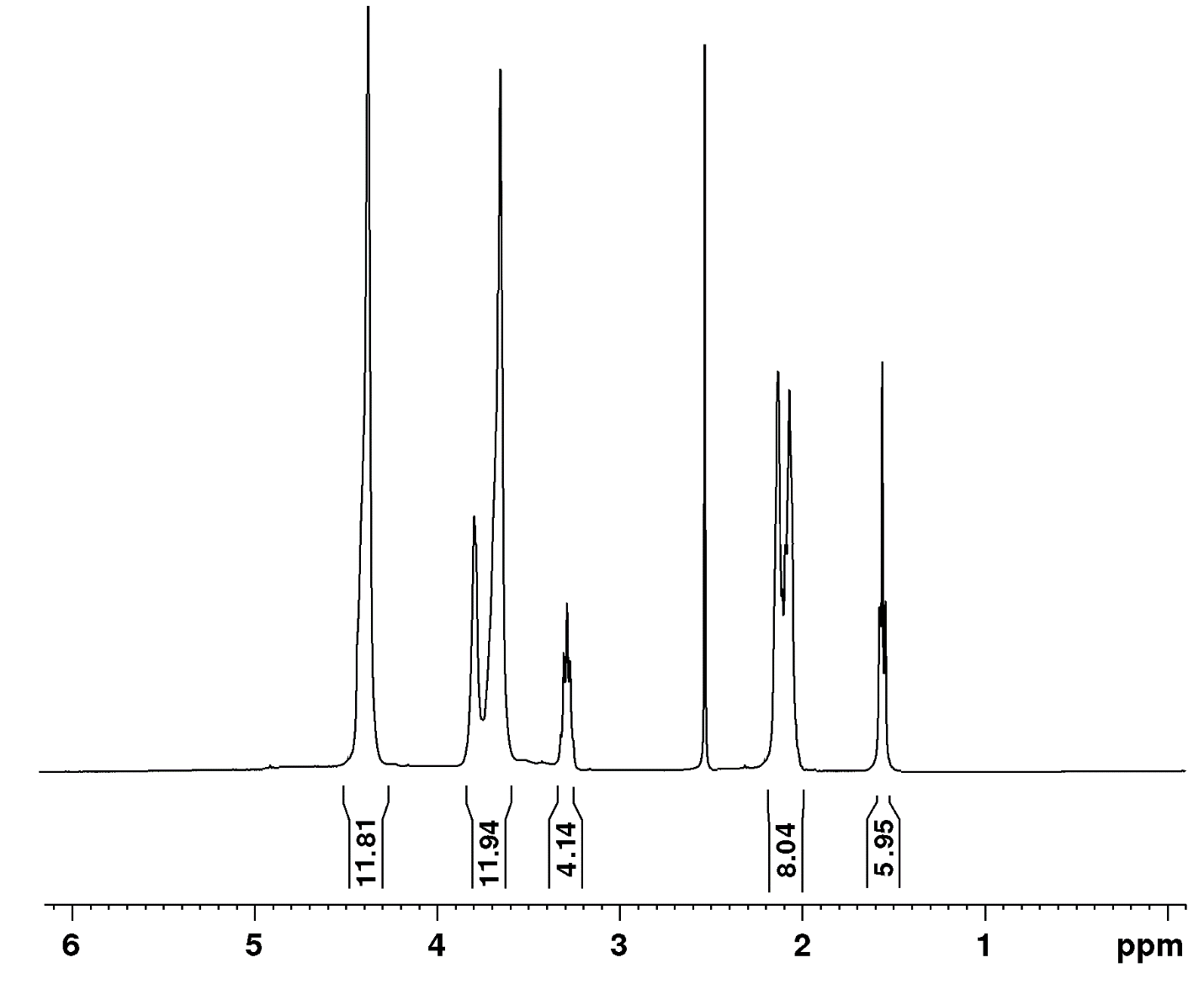


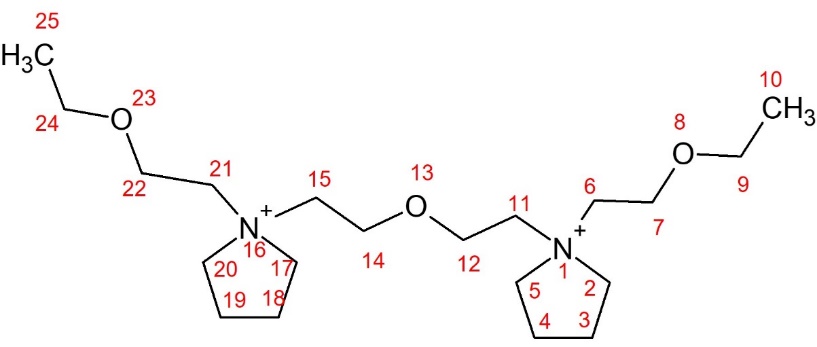


**Figure S2a.** ^1^H NMR spectra of (C_2_OC_2_Pyr-C_2_OC_2_-C_2_OC_2_Pyr)[TFSI]_2_

^1^H (d_6_-DMSO): 1.53 (6H, C11 and C27), 2.05-2.18 (8H, C3, C4, C18 and C19), 3.25 (4H, C9 and C24), 3.61-3.87 (12H, C2, C5, C7, C17, C20 and C22), 4.36-4.54 (12H, C6, C11, C12, C14, C15, C21)


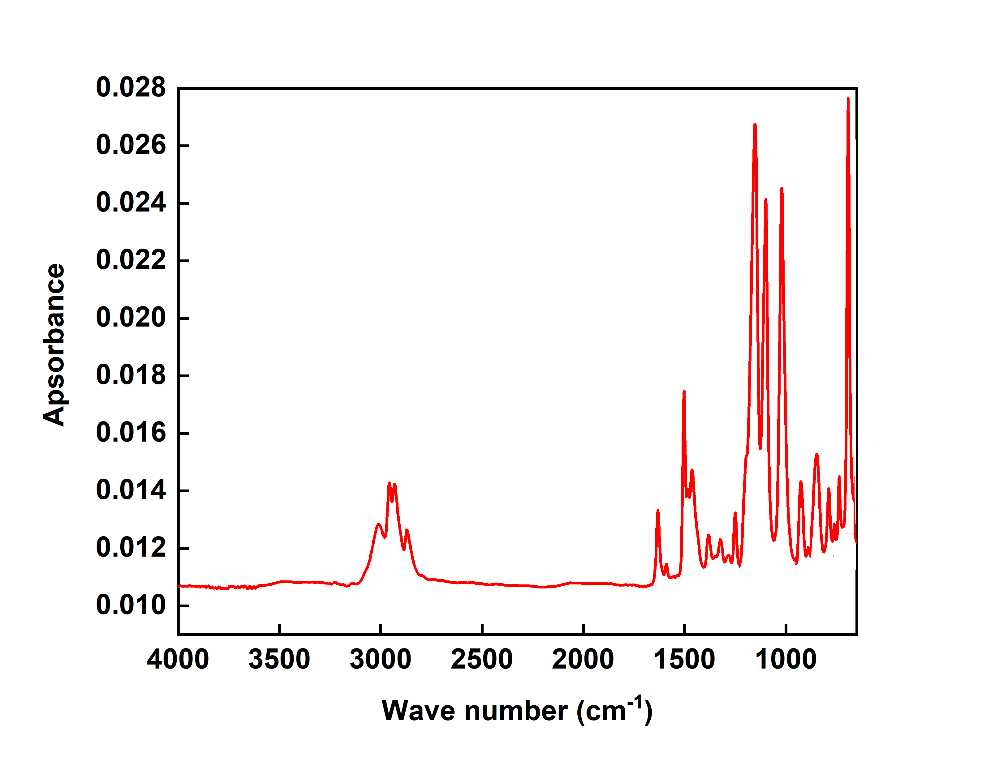


**Figure S2b.** FT-IR spectrum of (C_2_OC_2_Pyr-C_2_OC_2_-C_2_OC_2_Pyr)[TFSI]_2_

IR assignment: 3018 cm^-1^ C-H stretch mode in CH_3_; 2959, 2929 and 2870 cm^-1^ C-H stretch mode in CH_2_ of the pyrrolidinium ring; 1633 cm^-1^ N-C bend mode in the pyrrolidinium; 1466 cm^-1^ CH_3_ scissor mode (connected with the N-atom); 1207 cm^-1^ symmetric stretch mode of C-O-C; 1151 cm^-1^ symmetric stretch mode in SO_2_; 1125 cm^-1^ asymmetric strech mode of C-O-C; 910 cm^-1^ pyrrolidinium ring bend mode mode; 791 cm^-1^ stretch mode of S-N-S; 763 cm^-1^ stretch mode of C-S; 755 cm^-1^ stretch mode in CF_3_

**Figure S2c.** Mass spectrum of (C_2_OC_2_Pyr-C_2_OC_2_-C_2_OC_2_Pyr)[TFSI]_2_


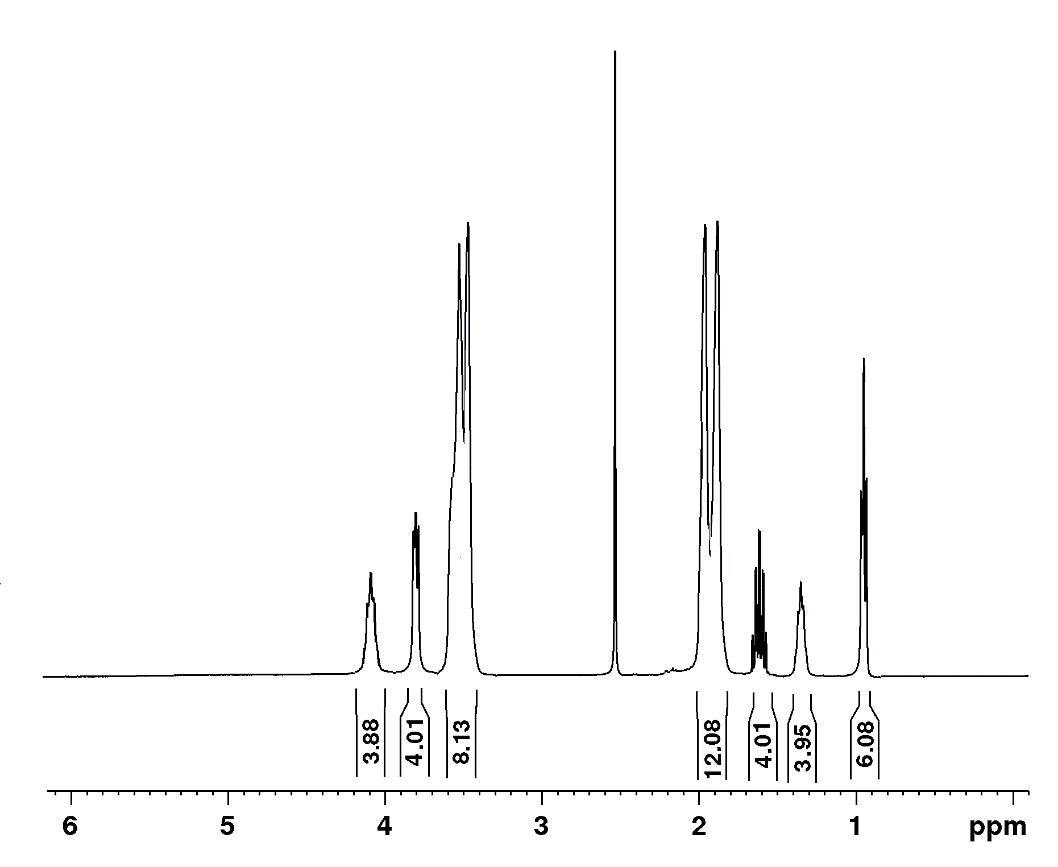


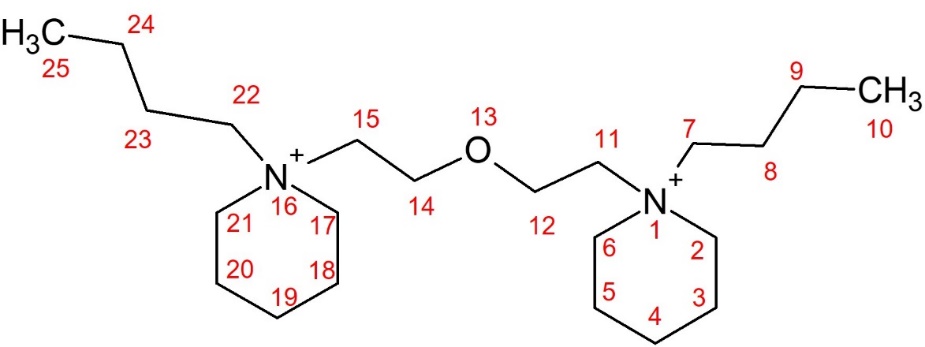


**Figure S3**. ^1^H NMR spectra of (C_4_Pip-C_2_OC_2_-C_4_Pip)[TFSI]_2_

^1^H (d_6_-DMSO): 0.92 (6H, C10 and C25), 1.36 (4H, C9 and C24), 1.58 (4H, C4 and C19), 1.84-2.01 (12H, C17, C27, C29, C40, C42, C45),3.52-3.66 (12H, C6, C26, C30, C39, C43, C44), 3.81 (4H, C33 and C37), 4.02 (4H, C34 and C36)


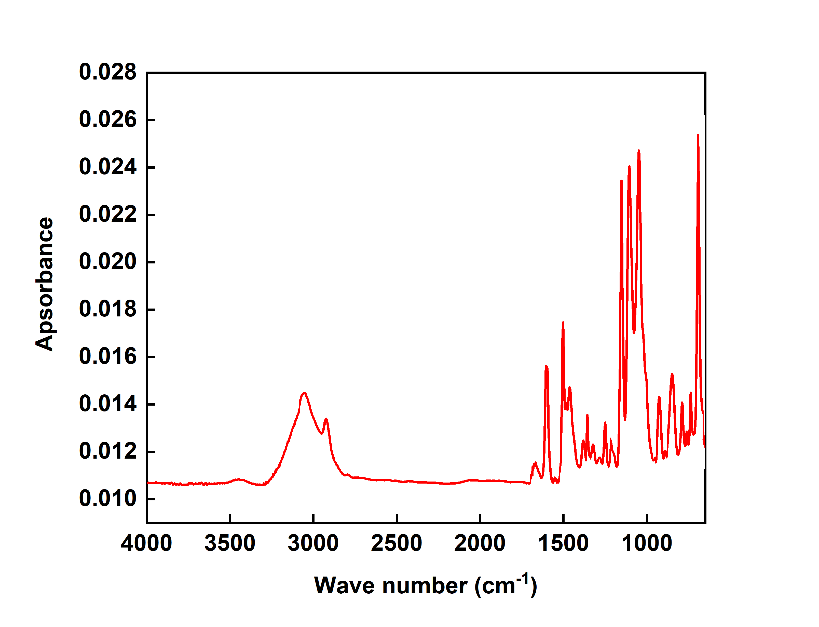


**Figure S3b.** FT-IR spectrum of (C_4_Pip-C_2_OC_2_-C_4_Pip)[TFSI]_2_

IR assignment: 3002 cm^-1^ asymmetric C-H stretch mode in the piperidinium ring; 2964 cm^-1^ symmetric C-H stretch mode in the piperidinium ring; 1552 cm^-1^ N-C bend mode in piperidinium; 1498 cm^-1^ CH_3_ scissor mode (connected with the N-atom); 1446 cm^-1^ asymmetric deformation of the butyl chain; 1198 cm^-1^ symmetric stretch mode of C-O-C; 1170 cm^-1^ symmetric stretch mode in SO_2_; 1131 asymmetric strech mode of C-O-C; 905 cm^-1^ piperidinium ring bend mode; 790 cm^-1^ stretch mode of S-N-S; 765 cm^-1^ stretch mode of C-S; 755 cm^-1^ stretch mode in CF_3_

**Figure S3c**. Mass spectrum of (C_4_Pip-C_2_OC_2_-C_4_Pip)[TFSI]_2_


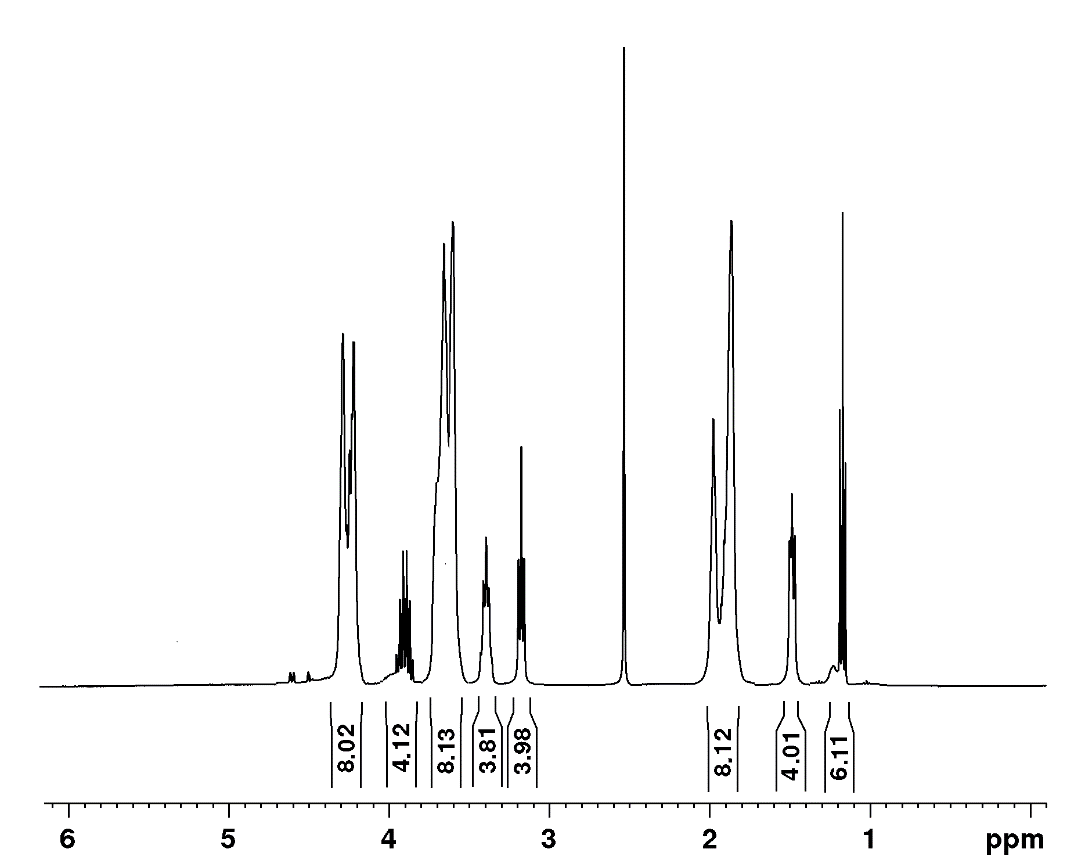


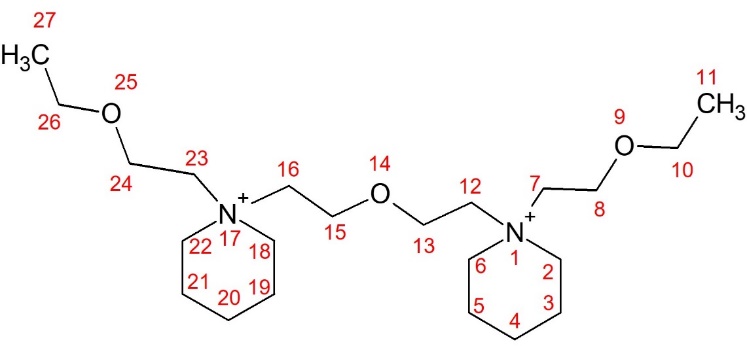


**Figure S4a**. ^1^H NMR spectra of (C_2_OC_2_Pip-C_2_OC_2_-C_2_OC_2_Pip)[TFSI]_2_

^1^H (d_6_-DMSO): 1.17 (6H, C11 and C27), 1,51 (4H, C4 and C20), 1.83-1.98 (8H, C3, C5, C19, C21), 3.19 (4H, C10 and C26), 3.34 (4H, C12 and C16), 3.58-3.72 (8H, C2, C6, C18 and C22), 3.83 (4H, C8 and C24), 4.26-4.41 (8H, C7, C13, C15 and C23)


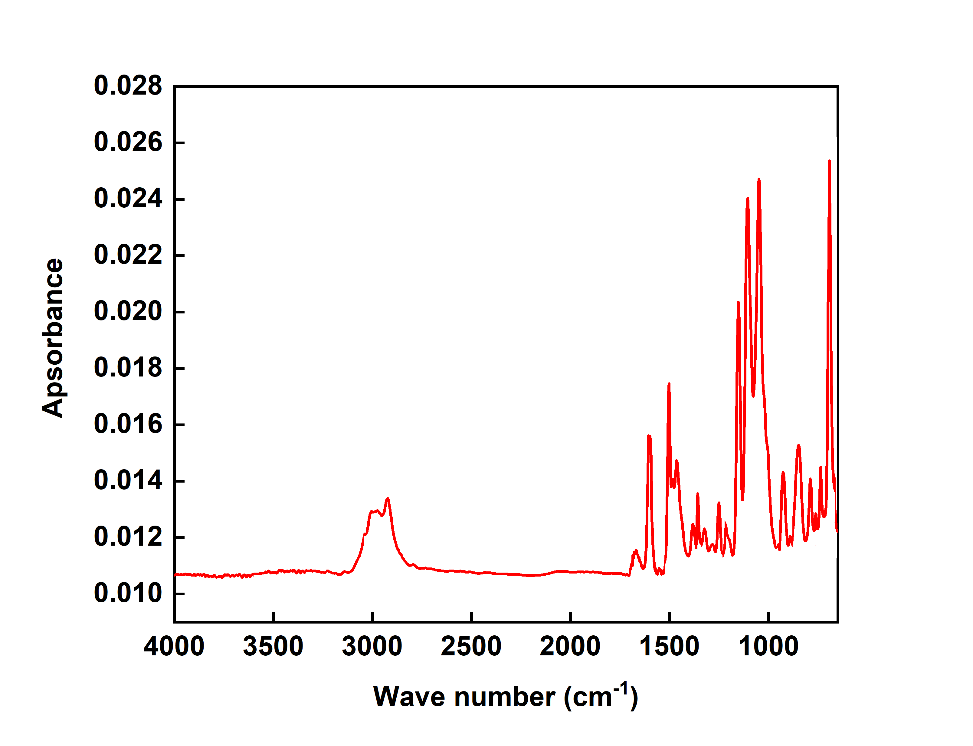


**Figure S4b.** FT-IR spectrum of (C_2_OC_2_Pip-C_2_OC_2_-C_2_OC_2_Pip)[TFSI]_2_

IR assignment: 2998 cm^-1^ asymmetric C-H stretch mode in the piperidinium ring; 2958 cm^-1^ symmetric C-H stretch mode in the piperidinium ring; 1563 cm^-1^ N-C bend mode in the piperidinium; 1523 cm^-1^ C-H bend mode; 1492 cm^-1^ CH_3_ scissor mode (connected with the N-atom); 1190 cm^-1^ symmetric stretch mode of C-O-C; 1164 cm^-1^ symmetric stretch mode in SO_2_; 1124 cm^-1^ asymmetric strech mode of C-O-C;

896 cm^-1^ piperidinium ring bend mode; 791 cm^-1^ stretch mode of S-N-S; 763 cm^-1^ stretch mode of C-S; 749 cm^-1^ stretchmode in CF_3_

**Figure S4c.** Mass spectrum of (C_2_OC_2_Pip-C_2_OC_2_-C_2_OC_2_Pip)[TFSI]_2_

a)

b)

**Figure S5.** Voltage profiles of the system Li[TFSI]_(1)_:(C_2_OC_2_Pyr-C_2_OC_2_-C_2_OC_2_Pyr)[TFSI]_2(1)_:H_2_O_(3)_; a) cycling experiments in coin cells, b) 1st cycle (solid line) with standard deviation (dotted line)

a)

b)

**Figure S6.** Voltage profiles of the system Li[TFSI]_(1)_:(C_4_Pyr-C_2_OC_2_-C_4_Pyr)[TFSI]_2(1)_:H_2_O_(3)_; a) cycling experiments in coin cells, b) 1st cycle (solid line) with standard deviation (dotted line)

a)

b)

**Figure S7.** Voltage profiles of the system Li[TFSI]_(1)_:(C_2_OC_2_Pip-C_2_OC_2_-C_2_OC_2_Pip)[TFSI]_2(1)_:H_2_O_(3)_; a) cycling experiments in coin cells, b) 1st cycle (solid line) with standard deviation (dotted line)

a)

b)

**Figure S8.** Voltage profiles of the system Li[TFSI]_(1)_:(C_4_Pip-C_2_OC_2_-C_4_Pip)[TFSI]_2(1)_:H_2_O_(3)_; a) cycling experiments in coin cells, b) 1st cycle (solid line) with standard deviation (dotted line)

**Raman-spectroscopic results.** A BioRad FTS 6000 spectrometer with a Raman accessory was used for recording the Raman spectra using a liquid nitrogen-cooled Ge detector. The effective frequency range studied was 500-4000 cm^-1^, and the spectral resolution was 4 cm^-1^.

**Figure S9.** The shift of the Raman S-N-S bend vibration mode with concentration. Black - Li[TFSI]_(1)_:(C_2_OC_2_Pyr-C_2_OC_2_-C_2_OC_2_Pyr)[TFSI]_2(1)_:H_2_O_(3)_; red - Li[TFSI]_(1)_:(C_4_Pyr-C_2_OC_2_-C_4_Pyr)[TFSI]_2(1)_:H_2_O_(3)_, blue – Li[TFSI]_(1)_:(C_2_OC_2_Pip-C_2_OC_2_-C_2_OC_2_Pip)[TFSI]_2(1)_:H_2_O_(3)_; green - Li[TFSI]_(1)_:(C_4_Pip-C_2_OC_2_-C_4_Pip)[TFSI]_2(1)_:H_2_O_(3)_; orange - Li[TFSI]_(1)_:H_2_O_(3)_.

**Molecular dynamics simulations.** MD simulations were performed using the Tinker software applying a polarizable force field developed by Borodin (O. Borodin, J.Phys. Chem. B, 2009, 113, 11463-11478). All simulated model systems were generated by replicating the Li[TFSI]-IL-H_2_O complexes using the xyzedit option in Tinker, which results in large simulation cells of 70-100 Å dimensions. The number of different molecular components in the simulation box was selected to match the experimental systems (50 molecules of lithium salt, 50 molecules of GIL, and 150 molecules of water). The simulation box dimensions were decreased in steps to 40-45 Å dimensions depending on the electrolyte composition during NPT-controlled equilibration runs at 333.15 K for 5-10 ns and with longer simulation times used for higher contents of water. Subsequently, the simulation temperature was reduced to 298.15 K and the electrolytes were equilibrated. The optimized electrolytes were further investigated by applying an NVT ensemble, using equilibrium box dimensions. The Ewald summation method was used to handle electrostatic interactions. Multiple timestep integration was employed with an inner timestep of 0.5 fs (bonded interactions); a central time step of 1.5 fs for all non-bonded interactions within a truncation distance of 7.0-8.0 Å and an outer time step of 3.0 fs for all non-bonded interactions between 7.0 Å and the non-bonded truncation distance of half of the simulation cell size. Each simulation run was at least 50 ns. The temperature and the pressure were controlled by A Nose-Hoover thermostat and a barostat. The equilibrium time was taken as the initial10 ns and was excluded from the analysis of data. In this work, only representative snapshots of systems in equilibrium are presented.


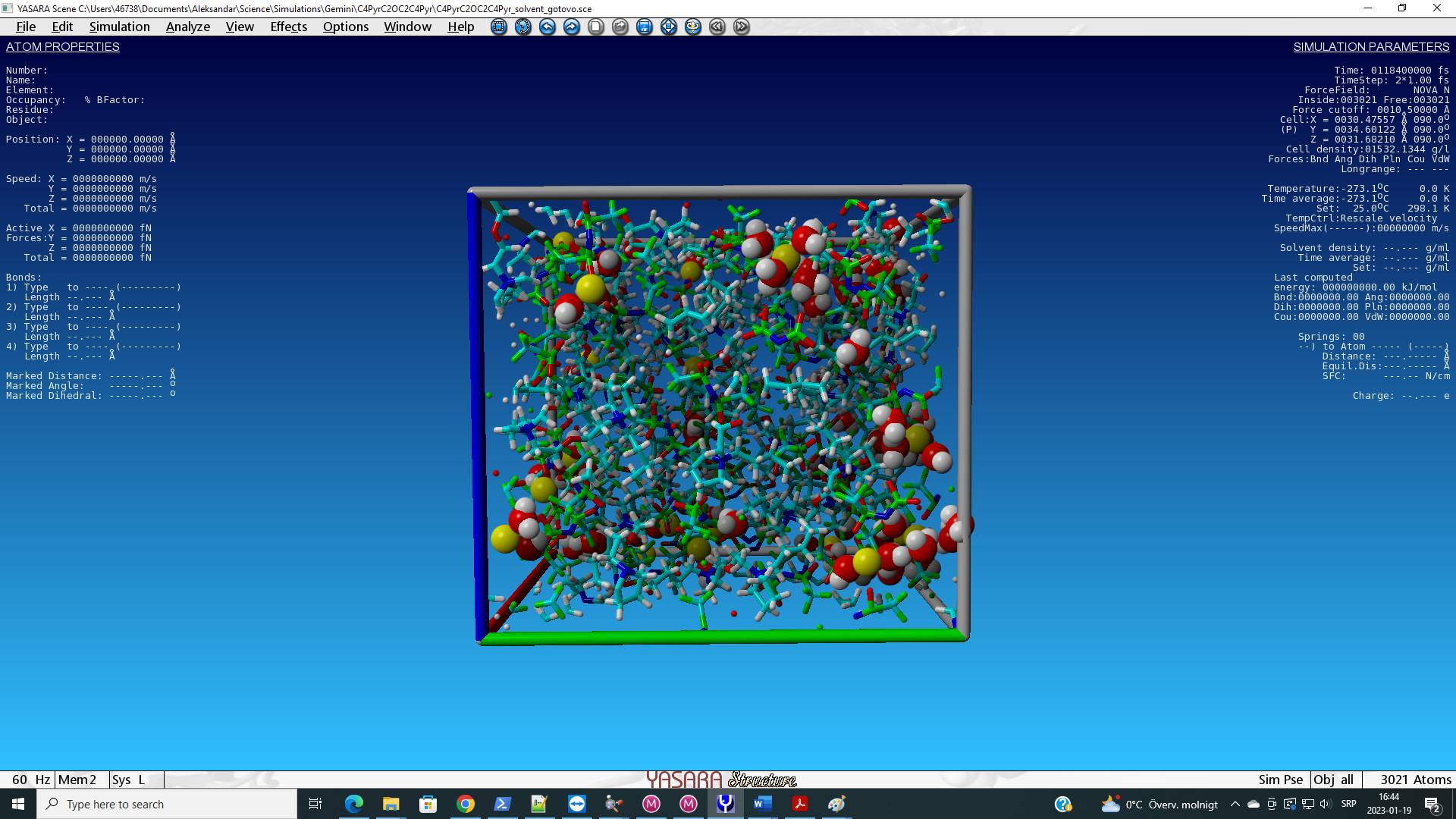

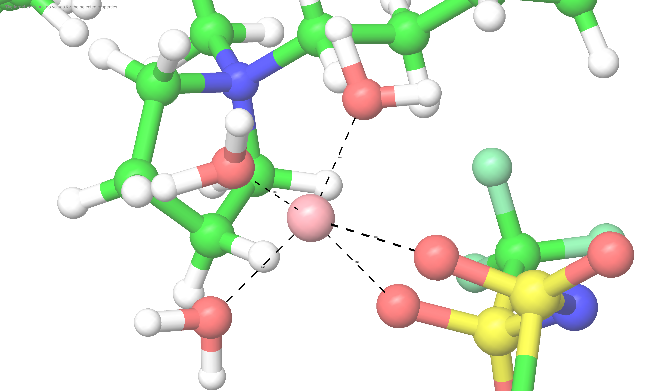


**Figure S10a.** MD simulation snapshot of Li[TFSI]_(1)_:(C_2_OC_2_Pyr-C_2_OC_2_-C_2_OC_2_Pyr)[TFSI]_2(1)_:H_2_O_(3)_. Left – a representative snapshot of the simulation box (lithium ions and water are shown as balls, while ionic liquid and anions are represented as sticks). Right – simulation environment around a lithium ion (displayed molecules within 5 Å of the lithium ion)


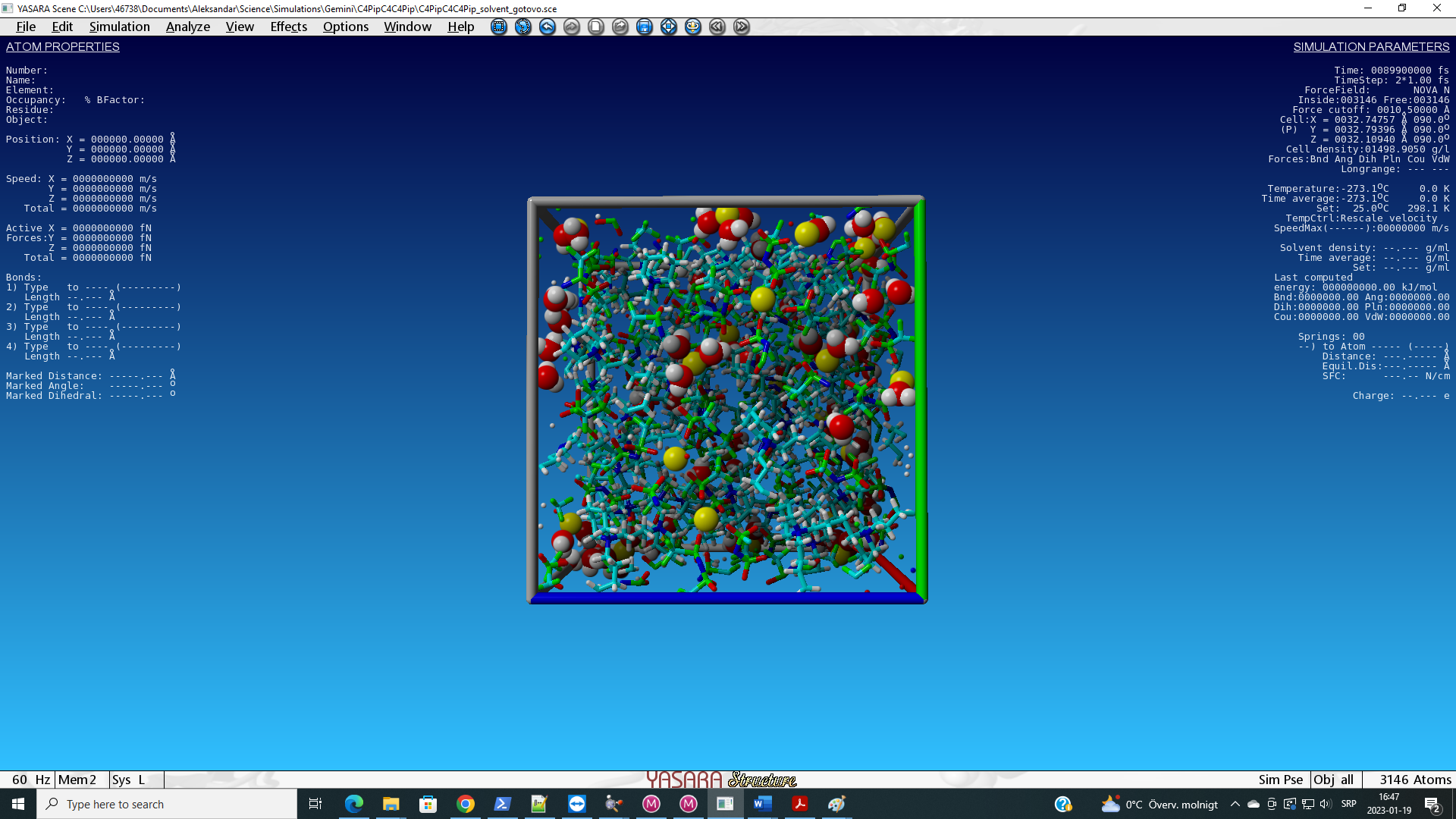

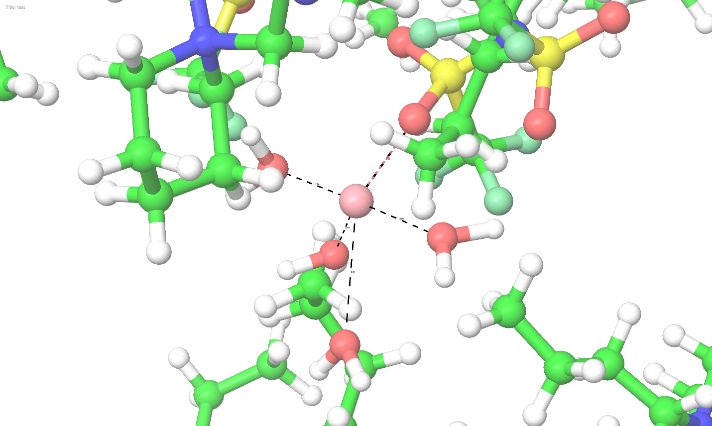


**Figure S10b.** MD simulation snapshot of Li[TFSI]_(1)_:(C_2_OC_2_Pip-C_2_OC_2_-C_2_OC_2_Pip)[TFSI]_2(1)_:H_2_O_(3)_. Left – a representative snapshot of the simulation box (lithium ions and water are shown as balls, while ionic liquid and anions are represented as sticks)). Right – simulation environment around a lithium ion (displayed molecules within 5 Å of the lithium ion)


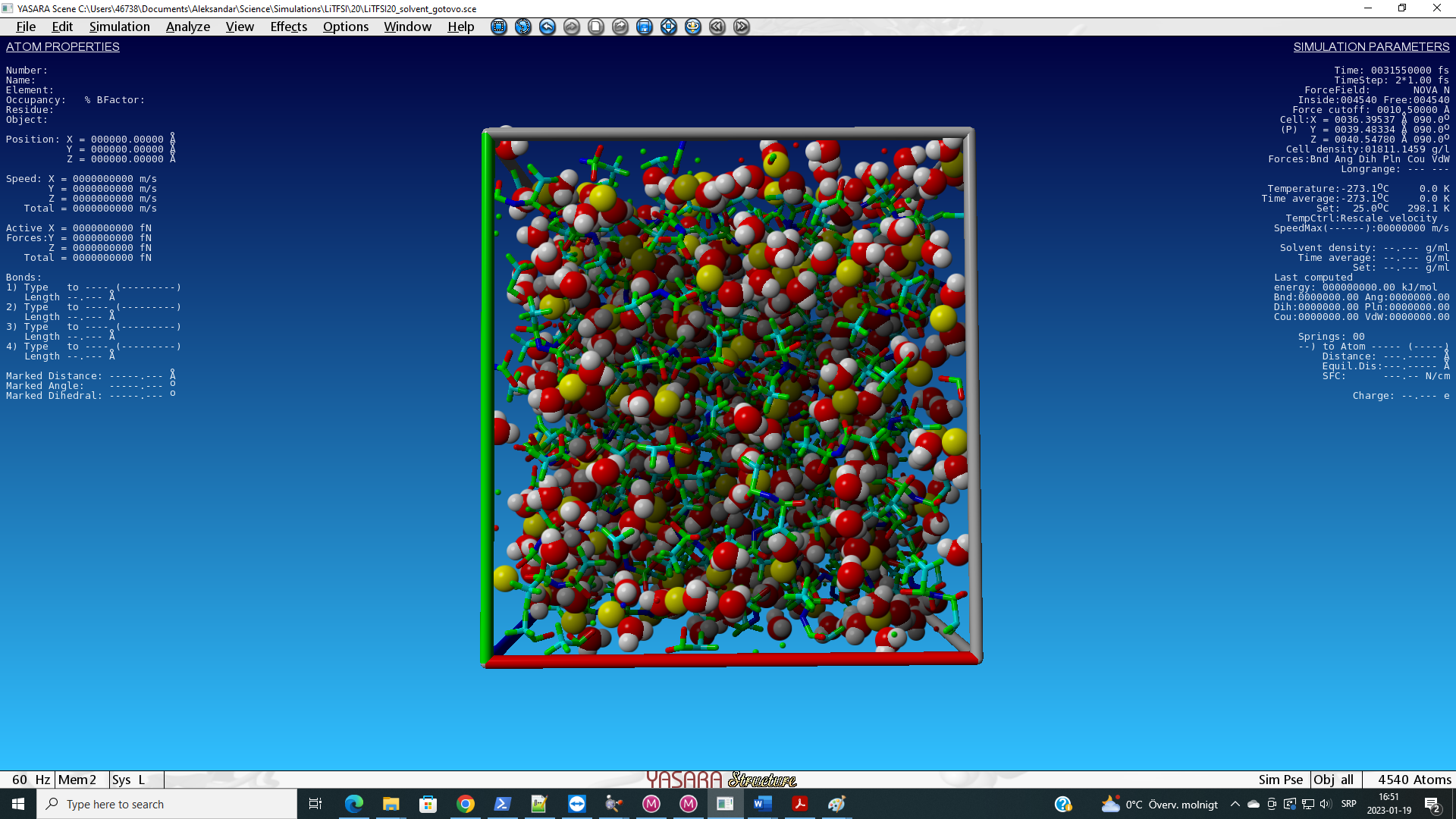

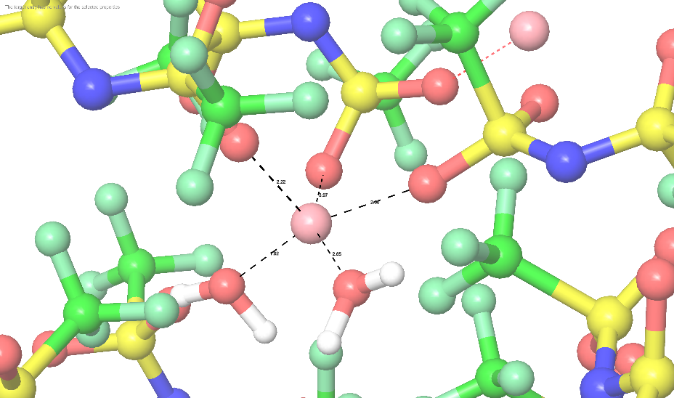


**Figure S10c.** MD simulation snapshot of Li[TFSI]_(1)_:H_2_O_(3)_. Left – a representative snapshot of the simulation box (lithium ions and water are shown as balls, while anions are represented as sticks)). Right – simulation environment around a lithium ion (displayed molecules within 5 Å of the lithium ion)
